# Phlda3 regulates beta cell survival during stress

**DOI:** 10.1038/s41598-019-49289-5

**Published:** 2019-09-06

**Authors:** Mohammed Bensellam, Jeng Yie Chan, Kailun Lee, Mugdha V. Joglekar, Anandwardhan A. Hardikar, Thomas Loudovaris, Helen E. Thomas, Jean-Christophe Jonas, D. Ross Laybutt

**Affiliations:** 1 Garvan Institute of Medical Research, Sydney, NSW Australia; 2 Université catholique de Louvain, Institut de recherche expérimentale et clinique, Pôle d’endocrinologie, diabète et nutrition, Brussels, Belgium; 3 St. Vincent’s Clinical School, UNSW Sydney, Sydney, NSW Australia; 4 NHMRC Clinical Trials Centre, The University of Sydney, Sydney, NSW Australia; 5 St. Vincent’s Institute of Medical Research, Fitzroy, Victoria Australia; 6 St. Vincent’s Hospital, The University of Melbourne, Fitzroy, Victoria Australia

**Keywords:** Molecular biology, Diabetes, Type 1 diabetes, Type 2 diabetes

## Abstract

The loss of functional beta cell mass characterises all forms of diabetes. Beta cells are highly susceptible to stress, including cytokine, endoplasmic reticulum (ER) and oxidative stress. This study examined the role of pleckstrin homology-like, domain family A, member 3 (*Phlda3*) in beta cell survival under stress conditions and the regulatory basis. We found that the mRNA levels of *Phlda3* were markedly upregulated *in vivo* in the islets of diabetic humans and mice. *In vitro*, exposure of MIN6 cells or islets to cytokines, palmitate, thapsigargin or ribose upregulated *Phlda3* mRNA and protein levels, concurrent with the induction of ER stress (*Ddit3* and *Trb3*) and antioxidant (*Hmox1*) genes. Furthermore, H_2_O_2_ treatment markedly increased PHLDA3 immunostaining in human islets. *Phlda3* expression was differentially regulated by adaptive (*Xbp1*) and apoptotic (*Ddit3*) unfolded protein response (UPR) mediators. siRNA-mediated knockdown of *Xbp1* inhibited the induction of *Phlda3* by cytokines and palmitate, whereas knockdown of *Ddit3* upregulated *Phlda3*. Moreover, knockdown of *Phlda3* potentiated cytokine-induced apoptosis in association with upregulation of inflammatory genes (*iNos*, *IL1β* and *IκBα*) and NFκB phosphorylation and downregulation of antioxidant (*Gpx1* and *Srxn1*) and adaptive UPR (*Xbp1*, *Hspa5* and *Fkbp11*) genes. Knockdown of *Phlda3* also potentiated apoptosis under oxidative stress conditions induced by ribose treatment. These findings suggest that *Phlda3* is crucial for beta cell survival under stress conditions. *Phlda3* regulates the cytokine, oxidative and ER stress responses in beta cells via the repression of inflammatory gene expression and the maintenance of antioxidant and adaptive UPR gene expression. *Phlda3* may promote beta cell survival in diabetes.

## Introduction

The loss of functional beta cell mass plays a crucial role in the pathogenesis of both type 1 and type 2 diabetes^1^. Compelling evidence has revealed that prolonged exposure to the (pre)diabetic milieu triggers beta cell stress and death. Indeed, inflammatory, endoplasmic reticulum (ER) and oxidative stress are potential mechanisms through which cytokines and elevated levels of glucose and free fatty acids induce beta cell apoptosis^2–6^. This has been associated with the activation of numerous proapoptotic effectors such as *Ddit3*, *Trb3*, *Txnip* and c-Jun N-terminal kinase^7–14^. However, stress stimuli also activate defense and adaptive responses, including the unfolded protein response (UPR) and the antioxidant response, that maintain homeostasis and promote beta cell survival^5,15,16^. The balance between protective and deleterious responses to stress determines beta cell fate, but the pathways involved are not fully characterized^5^. A better understanding of the beta cell stress response signaling pathways is needed to identify novel targets to preserve the functional beta cell mass in (pre)diabetic individuals.

*Phlda3* encodes for a member of the pleckstrin homology-like, domain family of proteins. The pleckstrin homology domain is an amino acid sequence of about 100 residues with a specific three-dimensional structure allowing binding to phosphoinositides and protein-protein interaction. It is present in a variety of proteins involved in signal transduction, phospholipid processing, membrane trafficking and organization of cytoskeleton^17^. The first member of this family, *Phlda1*, has previously been implicated in the modulation of energy metabolism and obesity^18^. On the other hand, *Phlda3* has been identified as a tumor suppressor in pancreatic neuroendocrine tumors^19^, and its expression is induced by ER stress in hepatocytes^20^. However, whether *Phlda3* expression is altered in diabetes or plays a role in beta cell pathophysiology are unclear.

In the present study, we report for the first time that *Phlda3* expression is upregulated in the islets of diabetic rodents and humans. Our findings in isolated islets and MIN6 beta cells suggest that *Phlda3* is induced in response to inflammatory, ER and oxidative stress and that it plays an important adaptive role during these stresses. Indeed, *Phlda3* knockdown potentiates inflammatory- and oxidative stress-induced apoptosis. Mechanistically, we demonstrate that the adaptive UPR effector *Xbp1* is required for *Phlda3* induction, whereas the pro-apoptotic effector *Ddit3* inhibits its expression. Moreover, we show that the *Phlda3-*mediated protection against stress involves the modulation of proinflammatory, adaptive UPR and antioxidant gene expression. Our results therefore suggest that *Phlda3* is a novel adaptive gene induced under conditions of stress that promotes beta cell survival.

## Material and Methods

### Reagents

Cytokines IL1β, IFNγ and TNFα were obtained from R&D Systems (Minneapolis, MN, USA). Ribose, thapsigargin and 4-hydroxytamoxifen were from Sigma (St. Louis, MI, USA). Control Non-Targeting and ON-TARGETplus SMARTpool siRNAs and transfection reagent DharmaFECT3 were from Thermo Fisher Scientific (Lafayette, CO, USA).

### Human islets

Human islets were obtained from 8 non-diabetic and 5 diabetic subjects at the Tom Mandel Islet Transplant Program in Melbourne^21^. Human islets were purified from heart-beating, brain-dead donors, with written informed consent from next of kin. All human studies were approved by the St Vincent’s Hospital Human Research Ethics Committee (approval number HREC011/04) and all methods were carried out in accordance with guidelines and regulations. Characteristics of organ donors and islet preparations are indicated in Supplementary Table 1. To evaluate the impact of oxidative stress on *Phlda3* protein expression *ex vivo*, human islets were obtained from 3 non-diabetic subjects through the JDRF award 31-2008-416 (ECIT Islet for Basic Research program) with written informed consent from next of kin and approved for use under the ethics reference B403/2017/05JUL/355 (Comité d'éthique hospitalo-facultaire Saint-Luc, UCLouvain). Characteristics of these donors and islet preparations are listed in Supplementary Table 2. All experiments were performed in accordance with relevant guidelines and regulations.

### Mice

14–16 weeks old C57BL/KsJ *db/db* mice and age-matched lean control mice (C57BL/KsJ), and 11–13 weeks old female nonobese diabetic (NOD) mice and age-matched control Balb/c mice were obtained from the Garvan Institute breeding colonies (Australian BioResources, Moss Vale, NSW, Australia). *Xbp1*^*flox/flox*^ mice were kindly provided by L.H. Glimcher and A.H. Lee (Weill Cornell Medical College, New York, NY, USA). They were crossed with *Pdx1*-*Cre*^*ER*^ mice to generate *Xbp1*^*+/+*^-*Pdx1*-*Cre*^*ER*^ (controls) and *Xbp1*^*flox/flox*^-*Pdx1*-*Cre*^*ER*^ mice. For *Xbp1* deletion, control and *Xbp1*^*flox/flox*^-*Pdx1*-*Cre*^*ER*^ islets were treated with 100 nmol/l 4-hydroxytamoxifen as previously described^14^. For *ex vivo* islet experiments, 8–10 week-old wild-type C57BL/6 J mice were used. All experiments were approved by the Garvan Institute/St. Vincent’s Hospital Animal Experimentation Ethics Committee and by the Institutional Committee on Animal Experimentation of the Health Sciences Sector at UCLouvain (Project 2017/UCL/MD/014). All experiments were performed in accordance with relevant guidelines and regulations.

### Islet isolation and culture

Islets were isolated by liberase digestion, separated by a density gradient and handpicked under a stereomicroscope. Islets were cultured in RPMI medium (Invitrogen, Carlsbad, CA, USA) containing 11.1 mmol/l glucose, 2 mmol/l glutamine, 10% heat-inactivated FBS, 50 units/ml penicillin and 50 µg/ml streptomycin.

### Cell culture

MIN6 beta cells (P26–43)^22^ were grown in Dulbecco’s modified Eagle’s medium (Invitrogen) containing 25 mmol/l glucose, 10 mmol/l HEPES, 10% FCS, 50 units/ml penicillin and 50 µg/ml streptomycin.

### Islet and cell treatment

Isolated islets and cells were treated with 100 U/ml IL1β, 250 U/ml IFNγ and 100 U/ml TNFα (15 min-24h). To assess the effects of lipotoxicity, islets and cells were treated with 0.92 g/100 ml BSA or 400 µmol/l palmitate coupled to 0.92 g/100 ml BSA (48 h). Thapsigargin (300 nmol/l, 1 µmol/l; 24 h) was used to induce ER stress. Ribose (50 mmol/l, 48 h) was used to induce oxidative stress. Cells were transfected with 100 nmol/l control, *Phlda3*, *Xbp1* or *Ddit3* siRNA using DharmaFECT3 transfection reagent following manufacturer’s instruction. Human islets were cultured in RPMI medium containing 5.5 mmol/l glucose in the absence or presence of 50 µmol/l H_2_O_2_ for 24 h.

### Apoptosis assay

Cell death was determined by quantification of cytoplasmic histone-associated DNA fragments using the Cell Death Detection ELISA (Roche Diagnostics, Castle Hill, NSW, Australia). Absorbance values were normalized to total DNA content measured by SYBR Green I (Roche Diagnostics, Castle Hill, NSW, Australia).

### RNA analysis

Total RNA was extracted using RNAeasy kit (Qiagen, Victoria, Australia) and cDNA synthesized using the QuantiTect reverse transcription kit (Qiagen, Victoria, Australia). Real-time RT-PCR was performed using power SYBR Green PCR Master Mix and a 7900HT Real-Time PCR system (Applied Biosystems, Foster City, CA, USA). Primer sequences are listed in Supplementary Table 3. The value obtained for a specific gene product was normalized to the control gene cyclophilin A and expressed as a fold-change of the value in control condition. For human samples, RNA was extracted using Trizol and cDNA was synthesized using a ‘High Capacity cDNA Reverse Transcription Kit’ as previously described^23^. Taqman gene expression assays were used for *Phlda3* (Hs00385313_ml) and the control gene *Gapdh* (Hs02758991_g1) (Applied Biosystems, Foster City, CA) using a TaqMan Fast Universal PCR Master Mix on a ViiA7 PCR machine.

### Protein analysis

Western blotting and band quantification were performed as previously described^24,25^. Phospho-v-akt murine thymoma viral oncogene homolog (AKT) (S473) and total AKT antibodies (9271 and 9272) were from Cell Signaling (Danvers, MA USA) and actin antibody (A2066) from Sigma. Activation of Nuclear factor κB (NFκB) was assessed by quantification of subunit p65 phosphorylation (pS536) using the NFκB p65 (pS536) SimpleStep ELISA kit (ab176647, Abcam, Cambridge, UK). Absorbance values were normalized to total protein content measured with the Pierce BCA protein assay kit (Thermo Fisher Scientific, Lafayette, CO, USA).

### Immunodetection of PHLDA3

After culture, cells on coverslips were washed with ice cold PBS and fixed in 4% paraformaldehyde for 15 min, permeabilized with PBS-Triton 0.05%, blocked with 5% BSA and incubated overnight at 4 °C with goat polyclonal anti-PHLDA3 antibody (ab22822, Abcam, Cambridge, UK) diluted 1:70 in 1% BSA. The next day, cells were washed and endogenous peroxidases inactivated with 3% H_2_O_2_ (vol/vol) before incubation for 1 h at room temperature with HRP-conjugated donkey polyclonal anti-goat secondary antibody (705-035-003, Jackson ImmunoResearch) diluted 1:500 in 1% BSA. Human islets were washed and fixed in 4% paraformaldehyde for 4 h and embedded in paraffin. Antigen retrieval was performed on 5 µm islet sections using a microwave in the presence of citrate buffer (pH 5,7). Islet sections were then treated with 3% H_2_O_2_ (vol/vol), blocked with 5% BSA and incubated overnight at 4 °C with goat polyclonal anti-PHLDA3 antibody diluted 1:100 in 1% BSA. The next day, islet sections were incubated for 1 h at room temperature with HRP-conjugated secondary antibody diluted 1:500 in 1% BSA. For cells and islet sections, the signal was revealed by 3,3’-diaminobenzidine (K3468, DAKO, Carpintera, USA). Islet sections were counterstained with hematoxylin (S3301, DAKO). Insulin and glucagon immunostainings were performed on adjacent islet sections as previously reported^24,25^. Anti-insulin antibody (3014, Cell Signaling Technology, Danvers, MA, USA) was diluted 1:500 and anti-glucagon antibody (G2654, Sigma) was diluted 1:2000. Secondary Alexa fluor antibodies (Thermo Fisher Scientific) were diluted 1:1000.

### Statistical analysis

Results are means ± SEM for the indicated number of experiments. Statistical significance was assessed by unpaired two-tailed student t-test, one-way ANOVA and a post-test of Newman-Keuls or two-way ANOVA and a post-test of Bonferroni.

## Results

### *Phlda3* mRNA levels are upregulated in the islets of diabetic mice and humans

**Figure 1:**
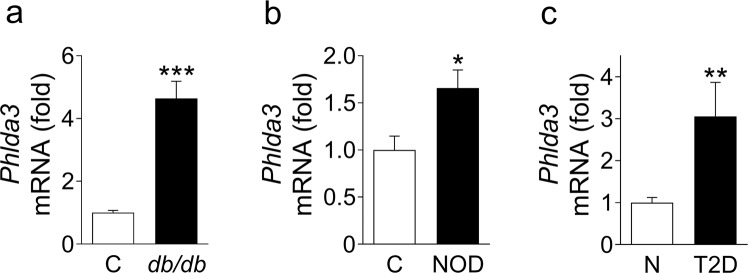
*Phlda3* mRNA levels are upregulated in the islets of diabetic animal models and human subjects. Changes in the mRNA levels of *Phlda3* in the islets of (**a**) control (C, white bar) and *db/db* mice (black bar), (**b**) control (C, white bar) and NOD mice (black bar) and (**c**) human non-diabetic (N, white bar) and type 2 diabetic (T2D, black bar) subjects. n = 6 animals per group for (**a**), n = 5–6 animals per group for (**b**) and n = 5–8 human subjects per group for (**c**). *p < 0.05, **p < 0.01, ***p < 0.001 vs control animals or non-diabetic subjects.

We first assessed whether the *Phlda3* expression is altered in diabetes. The mRNA levels of *Phlda3* were markedly upregulated in the islets of *db/db* mice, a model of type 2 diabetes, in comparison to age-matched lean control C57BL/KsJ mice (Fig. 1a). In NOD mice, a model of type 1 diabetes, we found a significant upregulation of *Phlda3* mRNA levels in comparison to control Balb/c mice (Fig. 1b). In humans, the mRNA levels of *Phlda3* were markedly upregulated in the islets of type 2 diabetes donors in comparison to control subjects (Fig. 1c). These findings show for the first time that the expression of *Phlda3* is upregulated in islets from diabetic rodents and humans. The findings raise two important questions: (1) what is (are) the mechanism(s) of *Phlda3* induction in beta cells? and (2) what is the role(s) of *Phlda3* in beta cell pathophysiology?

### *Phlda3* expression is induced by cytokine and palmitate treatment

**Figure 2:**
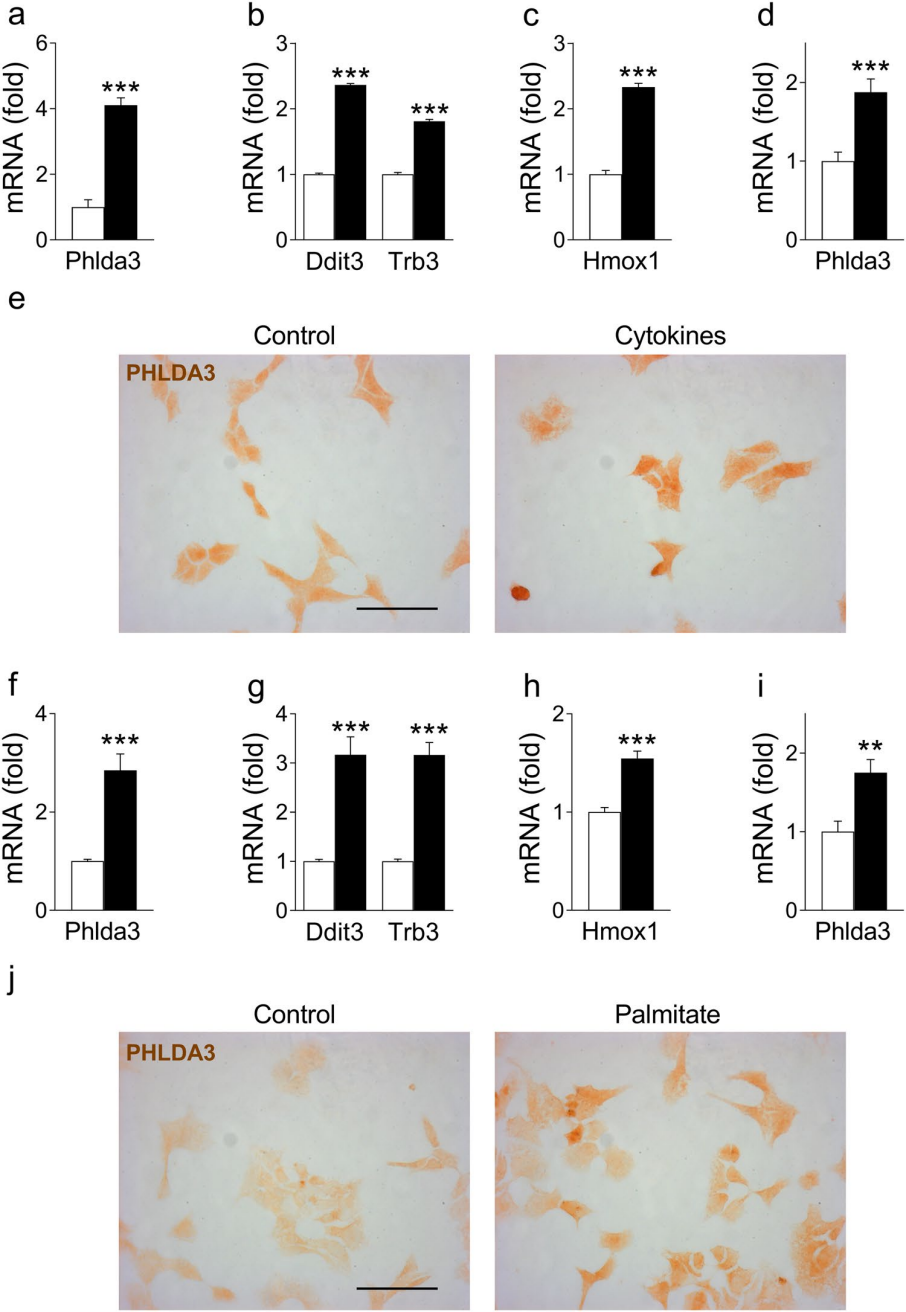
*Phlda3* mRNA and protein levels are upregulated by cytokine and palmitate treatment in parallel with the induction of ER stress and antioxidant genes. MIN6 cells (n = 4–9 experiments) or primary mouse islets (n = 5–10 experiments) were cultured in the absence (white bars) or presence (black bars) of cytokines (24 h) or palmitate (48 h). Changes in the mRNA levels of *Phlda3*, *Ddit3*, *Trb3* and *Hmox1* and PHLDA3 immunostaning in cytokine- (**a**–**c**,**e**) or palmitate-treated (**f**–**h**,**j**) MIN6 cells. Changes in the mRNA levels of *Phlda3* in cytokine- (**d**) or palmitate-high glucose-treated (**i**) islets. **p < 0.01, ***p < 0.001 vs control. Scale bars, 50 µm.

To explore the mechanism(s) underlying *Phlda3* induction in type 1 and type 2 diabetes, we exposed MIN6 cells and isolated mouse islets to factors that characterize the diabetic milieu, including proinflammatory cytokines and saturated free fatty acids^3,6^. We found that exposure of MIN6 cells to the cytokines IL1β, IFNγ and TNFα or the saturated fatty acid palmitate markedly upregulated the mRNA levels of *Phlda3* (Fig. 2a,f) with parallel induction of ER stress (*Ddit3*, *Trb3*) and antioxidant (*Hmox1*) genes (Fig. 2b,c,g,h). In agreement, PHLDA3 protein immunostaining was also increased by cytokine (Fig. 2e) and palmitate (Fig. 2j) treatments. These treatments have previously been demonstrated to be associated with increased beta cell death^14^. The upregulation of *Phlda3* mRNA levels by cytokine and palmitate treatments was confirmed in primary mouse islets (Fig. 2d,i). These results suggest that *Phlda3* is induced by common features of the diabetic environment in beta cells.

### *Phlda3* expression is induced by ER stress

**Figure 3:**
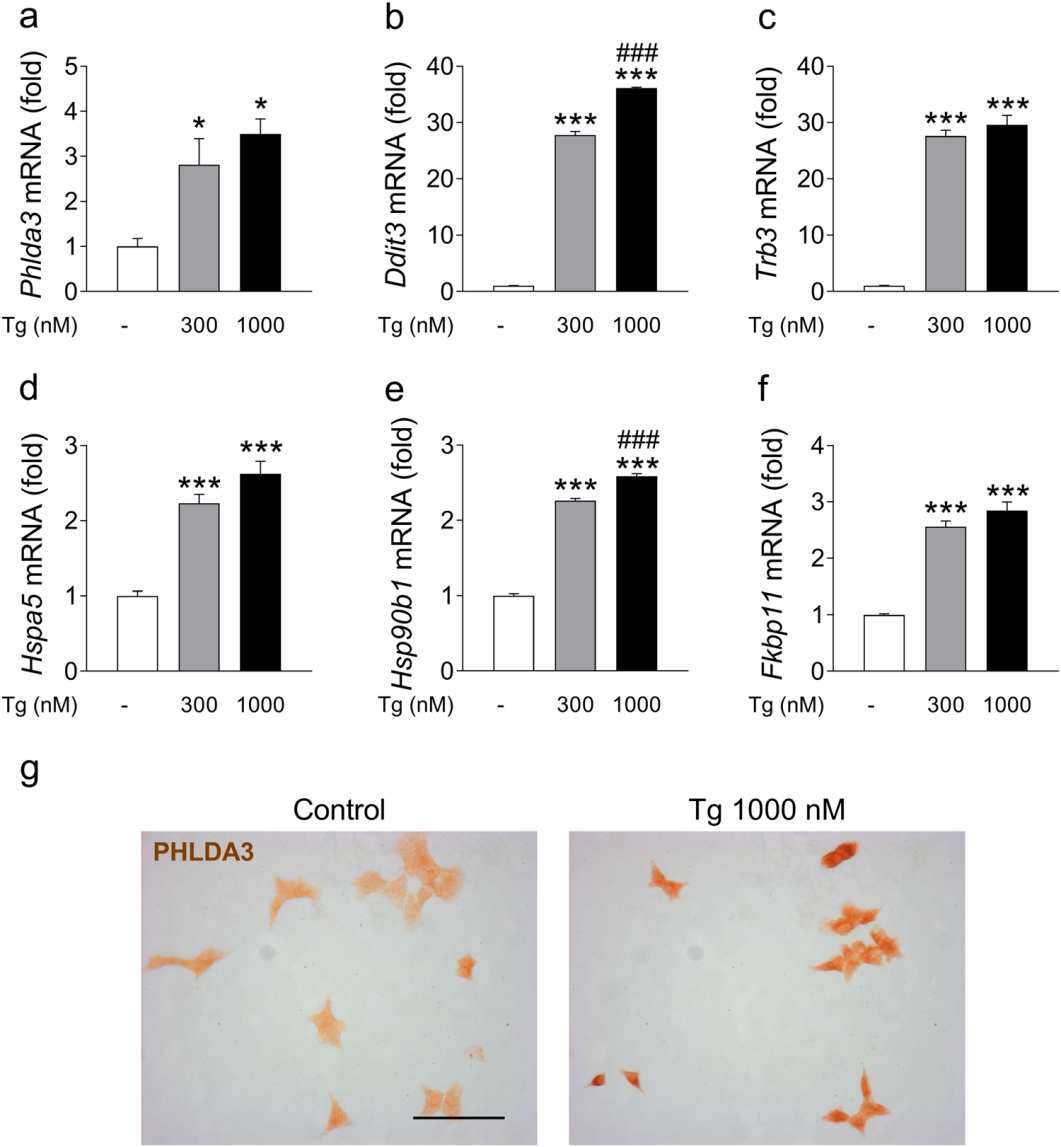
*Phlda3* mRNA and protein levels are upregulated by thapsigargin treatment in parallel with the induction of ER stress genes. MIN6 cells were cultured for 24 h in the absence (white bars) or presence of 300 nmol/l (grey bars) or 1000 nmol/l (black bars) thapsigargin (Tg). Changes in the mRNA levels of *Phlda3*, *Hmox1*, *Trb3*, *Hspa5*, *Hsp90b1* and *Fkbp11* (**a**–**f**) and PHLDA3 immunostaining (**g**). *p < 0.05, ***p < 0.001 vs control. ^###^*p* < 0.001 vs thapsigargin 300 nM. Scale bar, 50 µm.

ER stress is a key mechanism through which proinflammatory cytokines and palmitate have been shown to affect rodent and human beta cells^11,13,14,26,27^. We therefore tested whether exposure of beta cells to the pharmacological ER stress inducer thapsigargin affected *Phlda3* expression. Interestingly, *Phlda3* mRNA levels were markedly upregulated by thapsigargin treatment (Fig. 3a) in parallel with increased mRNA levels of adaptive (*Hspa5*, *Hsp90b1* and *Fkbp11*) and proapoptotic (*Ddit3* and *Trb3*) UPR genes (Fig. 3b–f). This finding was also confirmed at the protein level. Indeed, PHLDA3 protein immunostaining was markedly increased by thapsigargin treatment (Fig. 3g). These results demonstrate that *Phlda3* is a novel ER stress-responsive gene in beta cells.

### *Phlda3* expression is induced by oxidative stress

**Figure 4:**
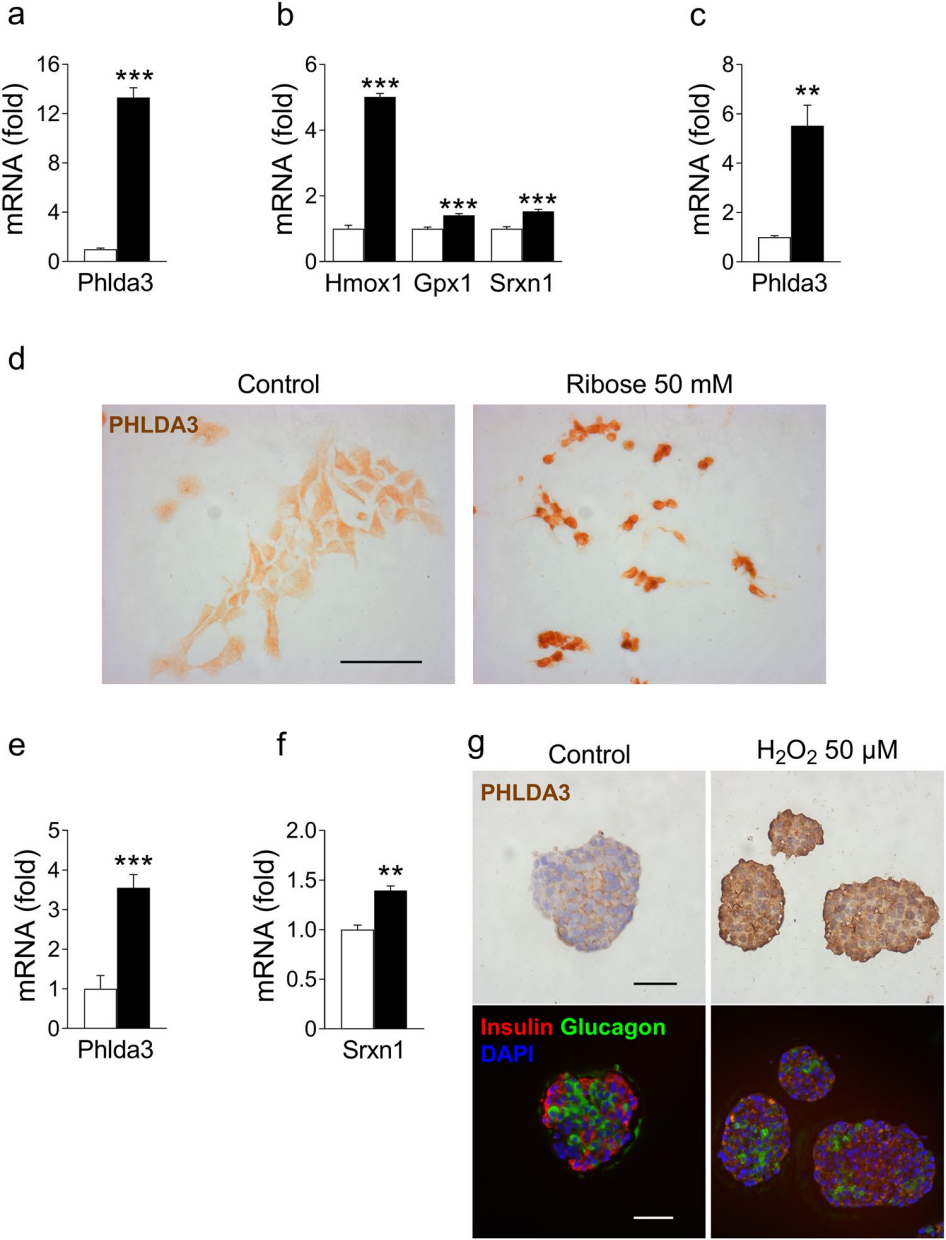
*Phlda3* mRNA and protein levels are upregulated by oxidative stress-inducing agents in parallel with the induction of antioxidant genes. MIN6 cells or primary mouse islets were cultured in the absence (white bars) or presence of 50 mmol/l ribose for 48 h or high glucose (30 mmol/l) for 3 weeks (black bars). Human islets were cultured in the absence or presence of 50 µmol/l H_2_O_2_. Changes in the mRNA levels of *Phlda3*, *Hmox1*, *Gpx1* and *Srxn1* in ribose-treated MIN6 cells (**a**,**b**). Changes in the mRNA levels of *Phlda3* in ribose-treated islets (**c**). Changes in PHLDA3 immunostaining in ribose-treated MIN6 cells (**d**). Changes in the mRNA levels of *Phlda3* and *Srxn1* in high glucose-treated islets (**e**,**f**). Changes in PHLDA3 immunostaining in H_2_O_2_-treated human islets and immunostaining for insulin and glucagon on adjacent islet sections (**g**). n = 3–5 experiments. **p < 0.01, ***p < 0.001 vs control. Scale bars, 50 µm.

Besides ER stress, cytokines and palmitate affect beta cells via the induction of oxidative stress. To determine the effects of oxidative stress on *Phlda3* in beta cells, we exposed MIN6 cells to ribose. Ribose is a sugar that produces ROS more potently than glucose and is an established model of beta cell glucotoxicity and oxidative stress^28,29^. Ribose treatment strongly upregulated *Phlda3* mRNA levels (Fig. 4a) with parallel upregulation of antioxidant genes (*Hmox1*, *Gpx1* and *Srxn1*) (Fig. 4b). We also confirmed upregulation of *Phlda3* mRNA levels by ribose treatment in primary mouse islets (Fig. 4c). In agreement, ribose treatment strongly increased PHLDA3 protein immunostaining in MIN6 cells (Fig. 4d). We next verified whether prolonged exposure to elevated glucose levels, thereby mimicking the diabetic milieu, may have an impact on *Phlda3* expression. Interestingly, culture of mouse islets for 3 weeks in the presence of 30 mmol/l glucose instead of 10 mmol/l markedly upregulated *Phlda3* mRNA levels (Fig. 4e) in parallel with the upregulation of the antioxidant gene *Srxn1* (Fig. 4f). Confirming these findings in humans, we found that exposure of human islets to H_2_O_2_ strongly upregulated PHLDA3 immunostaining (Fig. 4g and Supplementary Fig. 1). Interestingly, insulin and glucagon immunostaining on adjacent islet sections revealed that PHLDA3 protein expression was induced throughout the islets in beta cells as well as in islet non-beta cells including alpha cells (Fig. 4g).

Altogether, these results demonstrate that, in addition to ER stress, *Phlda3* is an oxidative stress responsive gene in beta cells.

### *Phlda3* is induced downstream of XBP1

**Figure 5:**
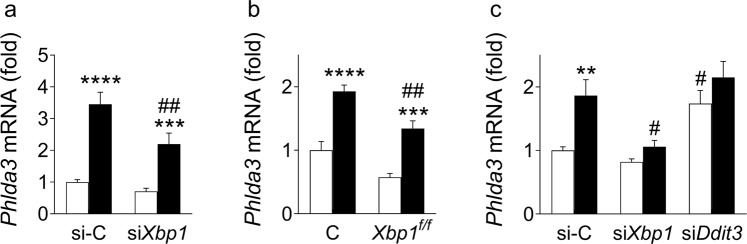
*Phlda3* induction under ER stress is downstream of XBP1. 4-Hydroxytamoxifen-treated control (C) and *Xbp1*^*flox/flox*^-*Pdx1*-*Cre*^*ER*^ (*Xbp1*^*f/f*^) mouse islets (n = 7 experiments) and MIN6 cells transfected with either control siRNA (si-C), siRNA against *Xbp1* (si*Xbp1*) or siRNA against *Ddit3* (si*Ddit3*) (n = 9–12 experiments) were cultured in the absence (white bars) or presence (black bars) of cytokines (24 h) or palmitate (48 h). Changes in the mRNA levels of *Phlda3* in cytokine-treated MIN6 cells (**a**) and primary islets (**b**). Changes in the mRNA levels of *Phlda3* in palmitate-treated MIN6 cells (**c**). **p < 0.01, ***p < 0.001, ****p < 0.0001 vs untreated. ^#^*p* < 0.05, ^##^*p* < 0.01 vs control siRNA or control islets.

ER stress triggers several signaling cascades in beta cells to restore homeostasis or otherwise induce apoptosis. X-box Binding Protein 1 (XBP1) is a key ER stress-inducible transcription factor that modulates the expression of several adaptive UPR genes including chaperones, foldases and components of the ER-associated degradation machinery^14^. It has previously been reported that *Phlda3* expression is regulated by XBP1 in hepatocytes^20^. To determine whether *Phlda3* is regulated by XBP1 in beta cells, we used siRNA-mediated inhibition of *Xbp1* expression in MIN6 cells^14^. In cytokine-treated MIN6 cells, *Xbp1* inhibition partially prevented *Phlda3* mRNA induction (Fig. 5a). We also used islets from *Xbp1*^*flox/flox*^-*Pdx1*-*Cre*^*ER*^ mice^14^. *Phlda3* induction by cytokines was partially prevented in islets with beta cell specific XBP1 deficiency in comparison to control islets (Fig. 5b). Moreover, in the MIN6 cell model of lipotoxicity, *Xbp1* inhibition using siRNA almost completely abolished the palmitate-mediated upregulation of *Phlda3* mRNA (Fig. 5c). We have previously demonstrated that inhibition of *Xbp1* is associated with increased cytokine and palmitate-induced cell death^14^. Taken together, the studies demonstrate a novel association between *Xbp1-Phlda3* and protection against cytokine toxicity and lipotoxicity in beta cells. Conversely, siRNA-mediated inhibition of the proapoptotic UPR gene *Ddit3* significantly upregulated the mRNA levels of *Phlda3* under basal conditions and they tended to be increased after palmitate treatment (Fig. 5c).

Altogether, these results demonstrate that *Phlda3* induction in beta cells is differentially regulated by adaptive (*Xbp1*) and proapoptotic UPR (*Ddit3*) effectors under conditions of cytokine and lipotoxic stress.

### *Phlda3* induction protects against cell death during stress

**Figure 6:**
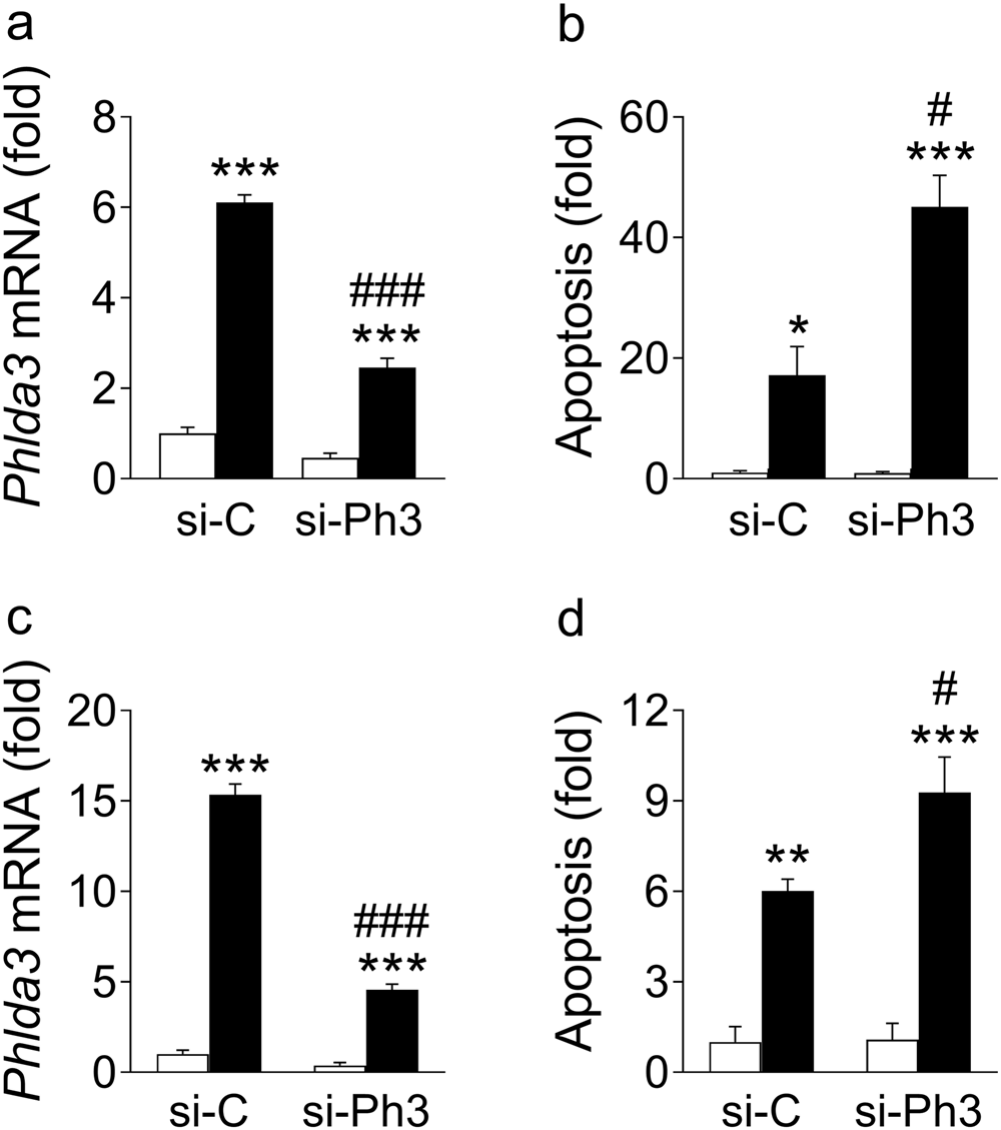
*Phlda3* knockdown potentiated cytokine- and ribose-induced apoptosis. MIN6 cells transfected with either control siRNA (si-C) or siRNA against *Phlda3* (si-Ph3) were cultured in the absence (white bars) or presence (black bars) of cytokines (24 h, **a**,**b**) or ribose (48 h, **c**,**d**). Changes in (**a**,**c**) mRNA levels and (**b**,**d**) apoptosis. n = 3–4 experiments. *p < 0.05, **p < 0.01, ***p < 0.001 vs untreated. ^#^*p* < 0.05, ^###^*p* < 0.001 vs control siRNA.

We next explored the role of *Phlda3* in stressed beta cells. To this end, we evaluated cell death under stress conditions after siRNA-mediated inhibition of *Phlda3* in MIN6 cells. Interestingly, we found that *Phlda3* knockdown (Fig. 6a) potentiated cytokine-induced apoptosis (Fig. 6b). Similarly, under oxidative stress conditions induced by ribose treatment, *Phlda3* knockdown (Fig. 6c) potentiated apoptosis (Fig. 6d). These results strongly suggest that *Phlda3* induction in beta cells under stress is adaptive and may play an important role to promote survival.

### *Phlda3* regulates several stress response pathways in beta cells

#### The NFκB pathway

**Figure 7:**
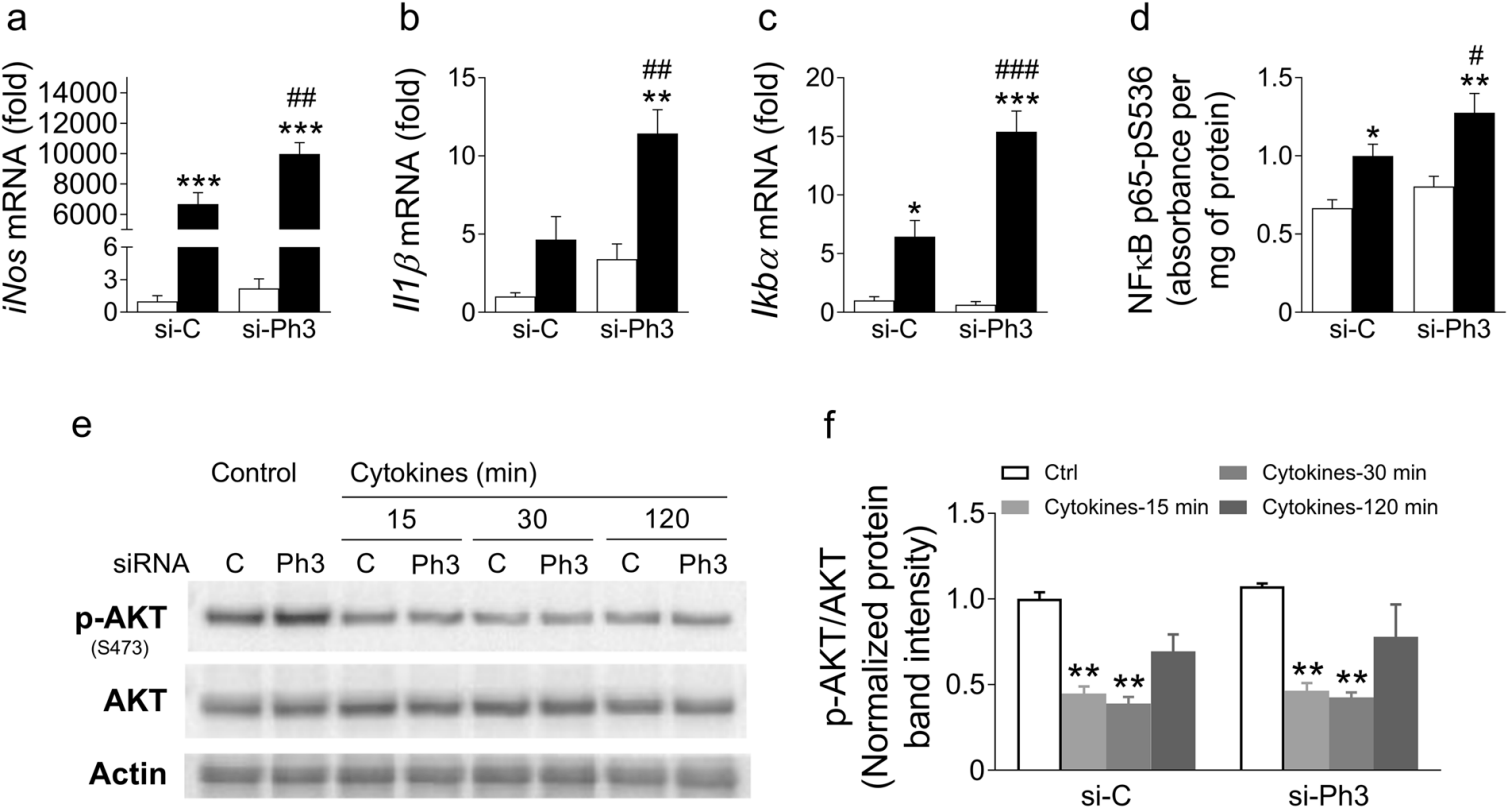
*Phlda3* inhibition potentiated cytokine-induced activation of the NFκB pathway independently of AKT. MIN6 cells transfected with either control siRNA (si-C) or siRNA against *Phlda3* (si-Ph3) were cultured in the absence (white bars) or presence (grey and black bars) of cytokines (15min-24h). Changes in the mRNA levels of *iNos*, *Il1*β and *Ikb*α (**a**–**c**). Changes in the phosphorylation of NFκB subunit p65 on serine 536 (**d**). Changes in phospho- and total AKT protein levels (**e**,**f**). n = 3–6 experiments. *p < 0.05, **p < 0.01, ***p < 0.001 vs untreated. ^#^*p* < 0.05, ^##^*p* < 0.01, ^###^*p* < 0.001 vs control siRNA.

*iNos* is an important effector implicated in cytokine-mediated beta cell death^26,30–32^. Interestingly, we found that *Phlda3* knockdown further increased *iNos* mRNA levels in control and cytokine-treated MIN6 cells (Fig. 7a). Since *iNos* is a downstream target of NFκB transcription factor^33–35^, we assessed the expression of *Il1*β and *I*κ*B*α, two other known NFκB target genes. Remarkably, the mRNA levels of both genes were further upregulated in cytokine-treated MIN6 cells after *Phlda3* knockdown (Fig. 7b,c). In agreement with these findings, cytokine-induced phosphorylation of NFκB subunit p65 on serine 536 (activation) was further increased after *Phlda3* knockdown (Fig. 7d). Taken together, these results strongly suggest that NFκB activation is potentiated upon *Phlda3* inhibition thereby leading to further upregulation of *iNos*, *Il1*β and *I*κ*B*α. Therefore, the protective effect of *Phlda3* induction under cytokines may stem, at least in part, from the repression of this pathway.

Evidence has suggested a role of the AKT pathway in NFκB activation in cancer cells^36–38^. Moreover, *Phlda3* has been proposed as a repressor of AKT^39^. Therefore, we assessed whether *Phlda3* inhibition affected AKT phosphorylation (activation) after cytokine treatment. Under basal conditions, differences in the phosphorylated AKT/AKT ratio were not detected in cells transfected with control or *Phlda3* siRNA (Fig. 7e,f). After cytokine treatment, AKT phosphorylation was rapidly and strongly reduced to a similar extent in cells transfected with control or *Phlda3* siRNA (Fig. 7e,f). These results argue against a potential contribution of AKT to the enhanced activation of the NFκB pathway observed after *Phlda3* inhibition.

#### The antioxidant response

**Figure 8:**
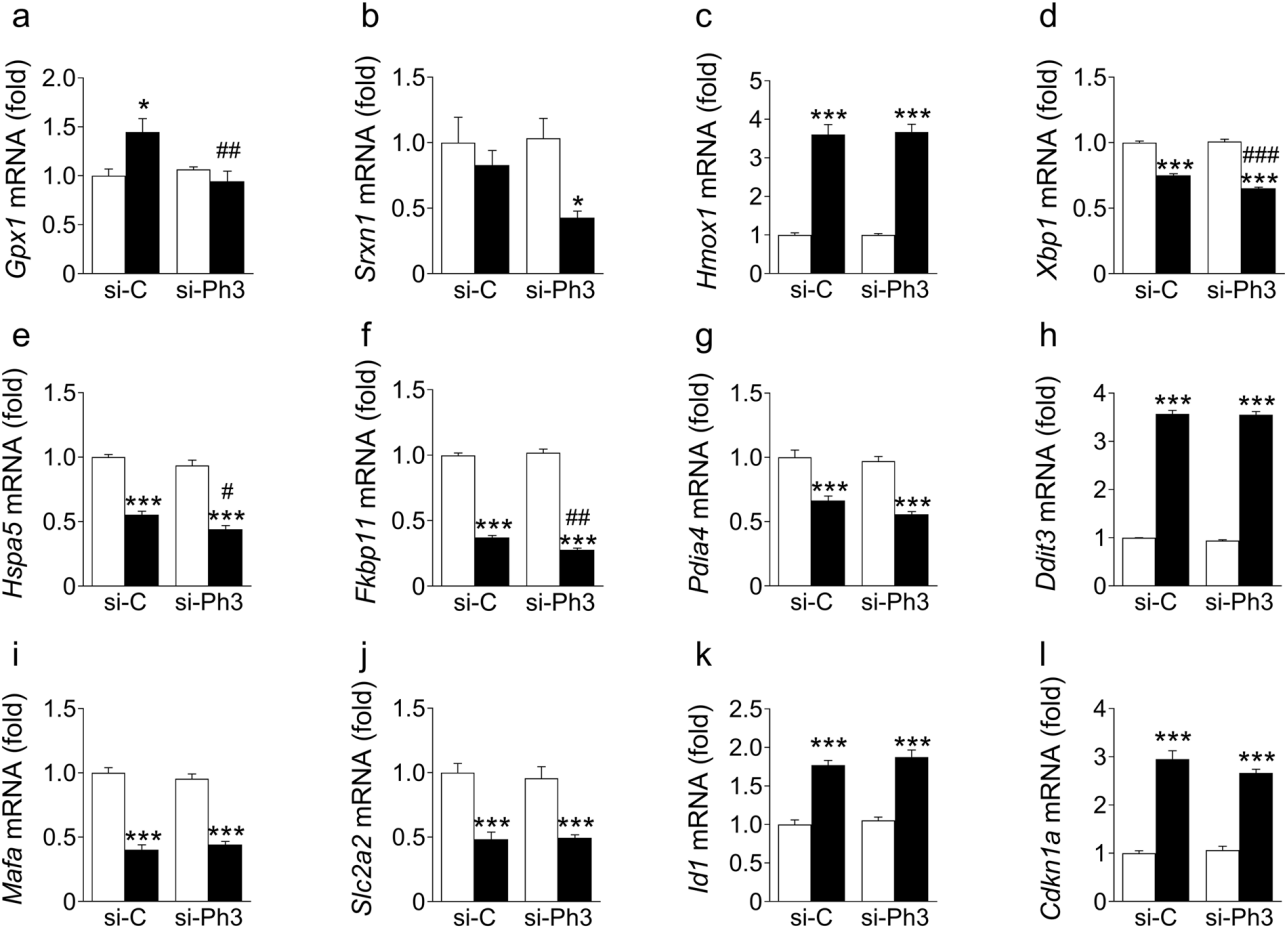
*Phlda3* inhibition negatively impacts antioxidant and adaptive UPR gene expression without affecting cytokine-induced alterations of beta cell differentiation. MIN6 cells transfected with either control siRNA (si-C) or siRNA against *Phlda3* (si-Ph3) were cultured in the absence (white bars) or presence (black bars) of cytokines (24 h). Changes in the mRNA levels of (**a**–**c**) antioxidant genes, (**d**–**g**) adaptive UPR gene, (**h**) *Ddit3*, (**i**,**j**) beta cell enriched genes and (**k**,**l**) genes involved in beta cell dedifferentiation. n = 4 experiments. *p < 0.05, ***p < 0.001 vs untreated. ^#^*p* < 0.05, ^##^*p* < 0.01, ^###^*p* < 0.001 vs control siRNA.

Since cytokine treatment induces oxidative stress in beta cells, we assessed whether the inhibition of *Phlda3* affected the antioxidant response. Interestingly, we found that cytokine-mediated upregulation of *Gpx1* mRNA levels was prevented after *Phlda3* inhibition (Fig. 8a). In addition, *Srxn1* expression was reduced in cytokine-treated cells after *Phlda3* inhibition (Fig. 8b). On the other hand, the mRNA levels of *Hmox1* were not affected (Fig. 8c). These results suggest that *Phlda3* is required for maintaining an adequate expression of specific antioxidant genes under stress conditions.

#### The adaptive UPR

We next assessed the influence of *Phlda3* inhibition on the ability of cytokines to downregulate the adaptive UPR. We found that the mRNA levels of adaptive UPR genes were significantly lower (*Xbp1*, *Hspa5*, *Fkbp11*) or tended to be lower (*Pdia4*) following cytokine treatment in cells transfected with *Phlda3* siRNA compared to control siRNA (Fig. 8d–g). On the other hand, the cytokine-mediated upregulation of the proapoptotic UPR gene *Ddit3* was not different between control siRNA- and *Phlda3* siRNA-treated cells (Fig. 8h). These results suggest that *Phlda3* partially protects against the loss of adaptive UPR gene expression during inflammatory stress.

Finally, we tested whether the inhibition of *Phlda3* influences cytokine-mediated beta cell dedifferentiation^40^. We found that the cytokine-mediated downregulation of beta cell genes *Mafa* and *Slc2a2* (also known as *Glut2*) was not affected by *Phlda3* knockdown (Fig. 8i,j). Furthermore, the cytokine-induced upregulation of genes associated with beta cell dedifferentiation, *Id1* and *Cdkn1a* (also known as *p21*) were not affected by *Phlda3* inhibition (Fig. 8k,l).

All together, these results demonstrate that *Phlda3* plays an important adaptive role under stress conditions via the modulation of several stress responses including the NFκB pathway, the antioxidant response and the UPR. Our studies suggest that *Phlda3* expression may be beneficial for preserving beta cell mass during the pathogenesis of diabetes.

## Discussion

**Figure 9:**
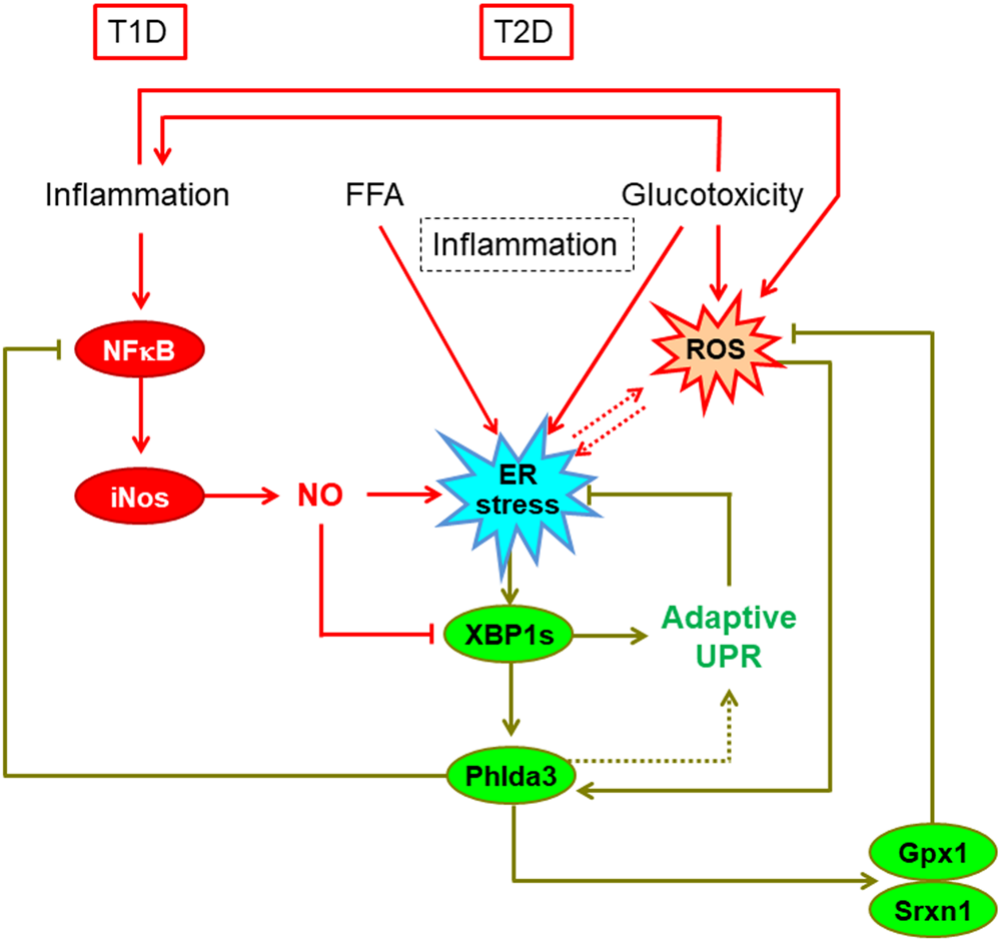
The proposed model. The elevated levels of proinflammatory cytokines, fatty acids and glucose play a key role in triggering beta cell stress and demise. *Phlda3* is a novel adaptive beta cell stress response gene induced downstream of XBP1 that promotes beta cell survival via the repression of the NFκB pathway and the maintenance of adequate antioxidant and adaptive UPR gene expression. FFA; free fatty acids, NO; nitric oxide, ROS; reactive oxygen species, T1D; type 1 diabetes, T2D; type 2 diabetes.

The elevated levels of fatty acids, glucose and proinflammatory cytokines of the (pre)diabetic environment play a major role in triggering beta cell stress and demise^3–5,41^. The complex interaction of stress-activated adaptive and proapoptotic responses determines the fate of beta cells. However, knowledge of the stress response pathways in beta cells is incomplete, which has hampered the development of strategies to preserve beta cell mass in diabetic subjects. In the present study, we have identified *Phlda3* as a novel stress-responsive gene in beta cells. We report for the first time that: (1) *Phlda3* is upregulated in islets of diabetic rodents and humans; (2) *Phlda3* expression is induced in response to the major stress conditions associated with beta cell failure in diabetes, namely inflammatory, ER and oxidative stress; and (3) *Phlda3* plays an important adaptive role to protect against beta cell death during stress (Fig. 9).

Beta cells are highly vulnerable to ER stress because of their heavy engagement in proinsulin biosynthesis. Therefore, an intact and fine-tuned adaptive UPR is vital for beta cell viability. Indeed, previous evidence linked the failure of this response with beta cell decompensation and progression to diabetes^42–44^. Interestingly, our data suggest that *Phlda3* plays a complex role both downstream and upstream of the XBP1 arm of the adaptive UPR (Fig. 9). Thus, we found that *Xbp1* was required for stress-induced upregulation of *Phlda3* in MIN6 cells and primary mouse islets. The *Xbp1*-dependent upregulation of *Phlda3* is in agreement with previous findings in hepatocytes^20^. However, in the latter study, *Phlda3* contributed to ER stress-mediated hepatocyte death^20^, in opposition to the protective role of *Phlda3*, and *Xbp1*^14^, in stressed beta cells. These findings are suggestive of distinct tissue-specific roles of *Xbp1*-*Phlda3* signalling during stress. Interestingly, inhibition of the proapoptotic ER stress gene *Ddit3* was associated with marked upregulation of *Phlda3* mRNA levels. This further supports the notion that *Phlda3* is protective in beta cells since *Ddit3* inhibition is associated with improved beta cell survival *in vitro*^14,25^ and *in vivo*^12^.

Moreover, our findings identify a previously unrecognized role of *Phlda3* as an upstream regulator of adaptive UPR gene expression. Thus, *Phlda3* inhibition reduced adaptive UPR gene mRNA levels after cytokine treatment (Fig. 8d–g). Interestingly, under these conditions *Phlda3* inhibition potentiated the upregulation of *iNos* (Figs 7a and 9). Notably, nitric oxide is a known repressor of the adaptive UPR^32^. Thus, changes in *iNos* activation may provide a mechanism whereby *Phlda3* regulates the adaptive UPR following cytokine stimulation.

In addition to *iNos*, *Phlda3* inhibition resulted in the upregulation of other NFκB target genes (*Il1*β and *Ikb*α) in parallel with increased NFκB phosphorylation. This raises the possibility that *Phlda3* acts as a brake on NFκB activation. Since the NFκB pathway is considered proapoptotic in beta cells^45^, the findings further support a protective role for *Phlda3*. How this repression may operate is unclear. We investigated AKT as a candidate because previous studies have linked it both with NFκB activation^36–38^ and repression by *Phlda3*^39^. However, in our model, the phosphorylation of AKT (activation) was unaffected by *Phlda3* inhibition under both basal conditions and following cytokine treatment (Fig. 7e,f). This suggests that the regulation of NFκB by *Phlda3* occurs independently of AKT. Alternatively, cytokine treatment triggers oxidative stress with subsequent upregulation of antioxidant genes. Oxidative stress can also upregulate the expression of inflammatory genes in beta cells^46^. Therefore, dysregulation of the antioxidant response such as occurs with *Phlda3* inhibition (reduced *Gpx1* and *Srxn1* mRNA levels, Fig. 8a,b) may lead to more severe oxidative stress. Accordingly, one could postulate that enhanced expression of NFκB target genes may result from increased oxidative stress under these conditions.

Our findings contrast with a recent report showing that islets from mice with *Phlda3* deletion are more resistant to hypoxic stress in the context of islet transplantation^47^. In our model, *Phlda3* knockdown had no significant effect on hypoxia-induced apoptosis (Supplementary Fig. 2). The reasons for these discrepant results are not clear. The studies of Sakata *et al*. adopted mice with constitutive whole body knockout of *Phlda3*. This raises the possibility of developmental effects in the knockout islets that are independent of *Phlda3* expression in adult beta cells. Indeed, the *Phlda3* knockout mice display a complex phenotype with the potential for metabolic changes in other tissues secondarily influencing islets. *Phlda3* knockout mice develop islet hyperplasia only later in life with an altered distribution of small and large islets. Perhaps *Phlda3* affects the stress response during beta cell ageing^48,49^, which may influence islet transplantation outcomes in the longer term^47^. Together with the knowledge that *Phlda1* regulates insulin sensitivity and energy expenditure^18^, it is clear that experiments that employ inducible beta cell specific modulation of *Phlda3* are needed. Moreover, hypoxic stress may involve unique regulatory networks compared with other stressors in beta cells, as exemplified by the strong suppression of XBP1 protein levels and downstream target genes^25^.

Interestingly, in mouse islets, *Phlda3* mRNA levels were markedly upregulated in the type 2 diabetes model (Fig. 1a), but displayed comparatively modest induction in the type 1 diabetes model (Fig. 1b). This may be related to differences in the nature or duration of the diabetes stress conditions between these models. Finally, the observation that oxidative stress upregulated PHLDA3 expression in both beta and non-beta cells in human islets suggests that PHLDA3 may have a potential role in islet non-beta cells, including alpha cells, which are also crucial for glucose homeostasis. Therefore, upregulated mRNA levels of *Phlda3* in human T2D islets may involve an effect in both beta and non-beta cells.

In conclusion, we have unveiled a novel role of *Phlda3* in beta cell pathophysiology. Our studies show for the first time that: (1) *Phlda3* is upregulated in the islets of diabetic mice and humans; (2) *Phlda3* is induced by inflammatory, ER and oxidative stress; (3) *Phlda3* is regulated positively by the adaptive UPR mediator, *Xbp1* and negatively by the proapoptotic UPR mediator, *Ddit3*; and (4) *Phlda3* contributes to the adaptive response to stress through modulation of the NFκB pathway, specific antioxidant genes and the adaptive UPR. These observations reveal a novel molecular mechanism regulating beta cell survival during stress and suggest the targeting of the *Xbp1*-*Phlda3* axis as a potential therapeutic strategy in diabetes.

## Supplementary information

Supplementary Tables and Figures
