# Supplementary material for: Phlda3 regulates beta cell survival during stress
Source: Sci Rep. 2019 Sep 6;9:12827. doi: 10.1038/s41598-019-49289-5 (PMC6731300; doi:10.1038/s41598-019-49289-5)

Supplementary information

Phlda3 regulates beta cell survival during stress

Mohammed Bensellam[1,2], Jeng Yie Chan[1,3], Kailun Lee[1], Mugdha V. Joglekar[4], Anandwardhan A. Hardikar[4], Thomas Loudovaris[5], Helen E. Thomas[5,6], Jean-Christophe Jonas[2] and D. Ross Laybutt[1,3]

[1] Garvan Institute of Medical Research, Sydney, NSW, Australia

[2] Université catholique de Louvain, Institut de recherche expérimentale et clinique, Pôle d'endocrinologie, diabète et nutrition, Brussels, Belgium

[3] St Vincent's Clinical School, UNSW Sydney, Sydney, NSW, Australia

[4] NHMRC Clinical Trials Centre, The University of Sydney, Sydney, NSW, Australia

[5] St Vincent's Institute of Medical Research, Fitzroy, Victoria, Australia

[6] St. Vincent's Hospital, The University of Melbourne, Fitzroy, Victoria, Australia

Corresponding authors:

Mohammed Bensellam

Université catholique de Louvain, Institut de recherche expérimentale et clinique, Pôle d'endocrinologie, diabète et nutrition, Brussels, Belgium.

Tel: 32-2-764-9586; Fax: 32-2-764-5532; Email: mohammed.bensellam@uclouvain.be

D. Ross Laybutt

Garvan Institute of Medical Research, 384 Victoria St, Darlinghurst, NSW 2010, Australia.

Tel: 61-2-9295-8228; Fax: 61-2-9295-8201; Email: r.laybutt@garvan.org.au

Supplementary Table 1. Characteristics of organ donors and islet preparations

Donor	Age	Gender	BMI	Diabetes duration	Diabetes treatment	Comments	Cause of death	Islet purity	Cell viability
N-SVI-014-10	34	M	30,6	-<br>HbA1c 5,4	-	No family history	Prescription drug overdose (28min downtime)	65%	74%
N-SVI-020-10	33	F	31	-<br>HbA1c 5,6	-	No family history	Suicide - Hanging (45min downtime)	80%	ND
N-SVI-032-10	40	F	24,1	-<br>HbA1c 5,7	-	No family history	Hypoxia (35min downtime)	90%	71%
N-SVI-042-10	65	M	36,8	-<br>HbA1c 5,7	-	No family history	Spontaneous subarachnoid hemorrhage	90%	ND
N-SVI-014-14	53	M	30,2	-	-	No family history	Cerebral hypoxia/ischemia (34min downtime)	70%	73%
N-SVI-023-14	61	F	31,9	-	-	No family history	Cerebral infarction (30min downtime)	90%	96%
N-SVI-040-14	39	M	23,9	-	-	Grandfather T2D	Cerebral hypoxia/ischemia (0 min downtime)	95%	83%
N-SVI-011-16	61	M	25,5	-	-	No family history	Intracranial haemorrhage	95%	84%
T2D- SVI-007-11	47	M	30,9	12 years<br>HbA1c 6.0	Diet controlled	Father T2D	Spontaneous subarachnoid hemorrhage	90%	85%
T2D- SVI-001-12	44	M	44,1	Undiagnosed<br>HbA1c 10,0	None	Alcoholic with liver cirrhosis, Father T2D on Dialysis. Not T1D no Autoantibodies	Spontaneous subarachnoid hemorrhage	70%	ND
T2D- SVI-005-14	57	F	24,8	<1 year	Diet/exercise controlled	Father, Uncle, Sister Insulin dependent Diabetics	Spontaneous subarachnoid hemorrhage	90%	ND
T2D- SVI-014-15	40	M	30,4	Undiagnosed<br>HbA1c 6,8	None	Mother T2D Confirmed not T1D no Autoantibodies	Hypoxia secondary to obstructive sleep apnea + community acquired pneumonia vs. meningoencephalitis (35min downtime)	75%	83%
T2D- SVI-010-16	65	M	37,2	15 years<br>HbA1c 8,0	Oral hypoglycemic, DIABEX (Metformin), DIAMICRON (Gliclazide)	Sister T2D	Cerebral infarction (27min downtime)	80%	ND

F: Female, M: Male, N: Non-diabetic, ND: Not determined, T2D: Type 2 diabetes

Supplementary Table 2. Characteristics of the three non-diabetic organ donors and their islet preparations

Donor	Age	Gender	BMI	Islet purity	Cell viability
1	62	F	29.3	90%	95%
2	58	M	27.2	80%	90%
3	50	F	28.8	95%	Not done

F: Female, M: Male

Supplementary Table 3. Sequences of oligonucleotide primers (5'-3')

Gene symbol	5' Oligonucleotide	3' Oligonucleotide
Cyclophilin (*Cypa, Ppia*)	TGTGCCAGGGTGGTGACTTTAC	TGGGAACCGTTTGTGTTTGG
Ddit3 (*Chop, Gadd153*)	TTCACTACTCTTGACCCTGCGTC	CACTGACCACTCTGTTTCCGTTTC
Dnajc3 (*p58^{IPK}*)	AAGCCCGTGGAAGCCATTAG	GGTCATTTTCATTGTGCTCCTGAG
Fkbp11	ACACGCTCCACATACACTACACGG	ATGACTGCTCTTCGCTTCTCTCCC
Gpx1	ACAGTCCACCGTGTATGCCTTC	CTCTTCATTCTTGCCATTCTCCTG
Glut2 (Slc2a2)	CATTCTTTGGTGGGTGGC	CCTGAGTGTGTTTGGAGCG
Hmox1(*HO-1*, *Hsp32*)	CCACACAGCACTATGTAAAGCGTC	GTTCGGGAAGGTAAAAAAAGCC
Hspa5 (*Bip*, *Grp78*)	AGGACAAGAAGGAGGATGTGGG	ACCGAAGGGTCATTCCAAGTG
Hsp90b1 (*Grp94*)	AAACGGCAACACTTCGGTCAG	GCATCCATCTCTTCTCCCTCATC
Id1	TTGGTCTGTCGGAGCAAAGC	GCAGGTCCCTGATGTAGTCGATTAC
Il1β	TGTTCTTTGAAGTTGACGGACCC	CCACAGCCACAATGAGTGATACTG
iNos (Nos2)	GCACCTTGGAAGAGGAGCAACTAC	TGCGGCTGGACTTTTCACTC
Ikbα (Nfkbia)	ACCCCTCTACATCTTGCCTGTGAG	CGTTGACATCAGCACCCAAAG
Mafa	CGTCAACGACTTCGACCTGATG	ATCCTCCAGCACCGCTTTTC
p21 (Cdkn1a, Waf1)	CTTGTCGCTGTCTTGCACTCTG	CTTCAGGGTTTTCTCTTGCAGAAG
Pdia4 (*Erp72*)	AGTCAAGGTGGTGGTGGGAAAG	TGGGAGCAAAATAGATGGTAGGG
Phlda3	CACATCTACTTCACGCTAGTGACCG	GTTGATTCTTGAACTTGACCAGGC
Srxn1	TACCAATCGCCGTGCTCATC	AAAGGAATAGTAGTAGTCGCCACCC
Trb3	TCTTCAGCAACTGTGAGAGGACG	TCCAGACATCAGCCGCTTTG
Xbp1	GCAGCAAGTGGTGGATTTGG	AGATGTTCTGGGGAGGTGACAAC

Aliases of gene symbols given in parentheses

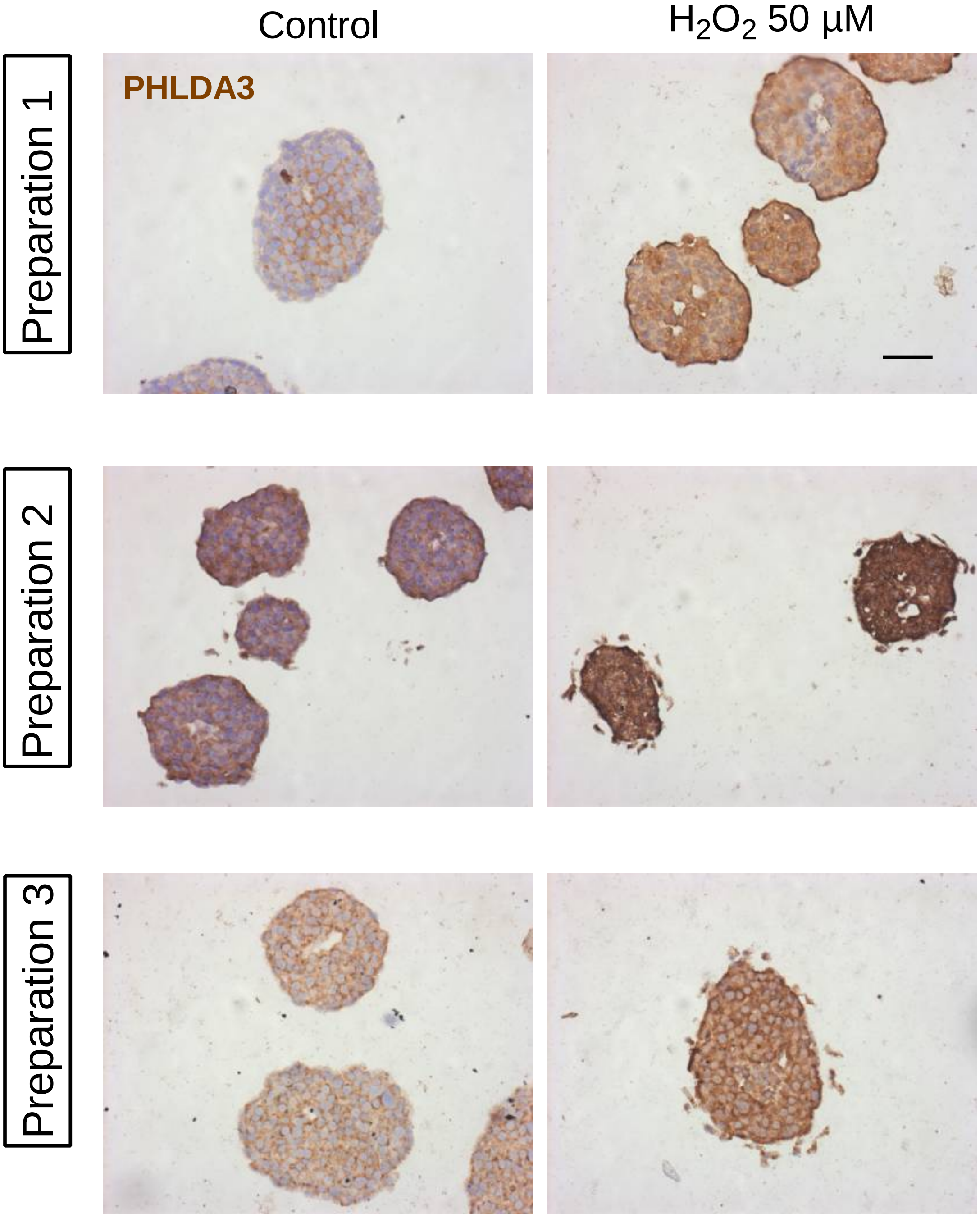

Supplementary Figure 1. PHLDA3 immunostaining in H_2O_2-treated human islets

Human islets from 3 different preparations were cultured in the absence or presence of 50 µmol/l H_2O_2 for 24h. At the end of culture, islets were fixed, embedded in paraffin and sections prepared for assessement of PHLDA3 expression. The representative images shown in Fig. 4g are from preparation 1. These images show the upregulation of PHLDA3 immunostaining throughout H_2O_2-treated islets in both beta and non-beta cells. Scale bar, 50 µm.

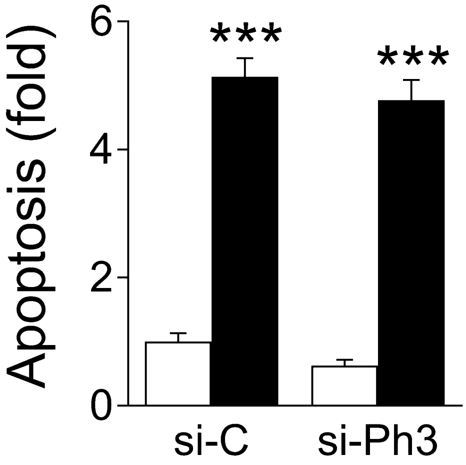

Supplementary Figure 2. Effect of *Phlda3* knockdown on hypoxia-induced apoptosis

MIN6 cells transfected with either control siRNA (si-C) or siRNA against *Phlda3* (si-Ph3) were cultured in the presence of normoxia (20% O_2; white bars) or hypoxia (1% O_2; grey bars) for 24h. Changes in apoptosis levels were normalized to DNA content. Data are means±SEM for 3 experiments. ***p<0.001 vs normoxia. (two-way ANOVA+test of Bonferroni).

Uncropped images of immunoblots used for experiments shown in figure 7e. The bands shown in figure 7e are indicated with horizontal arrows

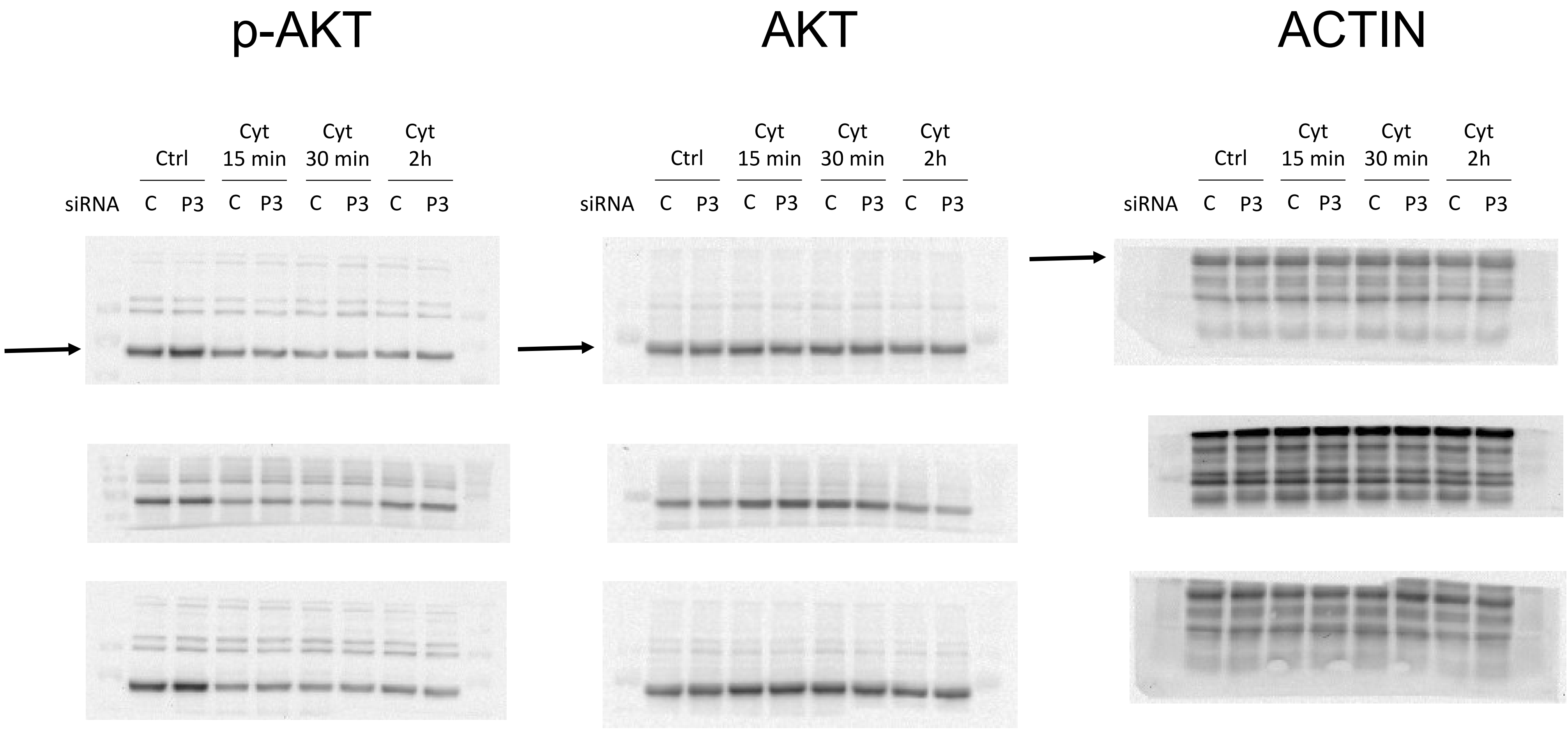

Supplement: Supplementary file 1 — Supplementary Tables and Figures [file 41598_2019_49289_MOESM1_ESM.pdf]
